# Supplementary material for: Direct Phenotyping and Principal Component Analysis of Type Traits Implicate Novel QTL in Bovine Mastitis through Genome-Wide Association
Source: Animals (Basel). 2021 Apr 17;11(4):1147. doi: 10.3390/ani11041147 (PMC8072530; doi:10.3390/ani11041147)
Supplement: Supplementary file 1 [file animals-11-01147-s001.zip › MilesA_Animals_U&T-GWAS_Supplementary-Table-1_v2.pdf]

Supplementary Table S1. QTL positions and candidate genes. Significantly associated QTL (Bonferroni < 0.05) with chromosome and base pair position, as well as potential candidate genes for all models are given. The implied role of each locus on the basis of their respective candidate genes, in either udder and teat morphology, mastitis immune responses, or other processes (N/A) is also summarized. Asterisks (*) indicate novel QTL.

Trait	Chromosome[1]	Position (bp)[1]	Candidate Genes[2]	Implied Role
Front Teat Length	10	50107685-50117321	*FOXB1, LOC112448464*	N/A
Front Teat Width	23	19946762	***PLA2G7,*** *ANKRD66, TRD6, SLC25A27*	immunology
	23	20566460-20576922	*TNFRSF21, CD2AP, ADGRF2*	morphology; immunology
Fore Udder Attachment	2	126354670-126359098	*WASF2, GPR3, CD164L2, FCN3, MAP3K6, SYTL1, TMEM222, WDTC1, SLC9A1, FAM46B, TRNP1, KDF1, NUDC, NR0B2, GPN2, SFN,* ***ZDHHC18****, PIGV, LOC112443408, ARID1A, RPS6KA1, HMGN2, LIN28A, ZNF683, CRYBG2, UBXN11, CEP85*	morphology; immunology
	X	121461599-121477381	*PHEX, LOC112445297, SMS, LOC112445297,* ***MBTPS2****, SMPX, KLHL34, TRNAC-GCA, LOC112445299, CNKSR2*	N/A
Risk PC1	15*	7287030*	*CEP126, ANGPTL5,* ***TRPC6***	morphology; immunology
Rear Teat Length	2	112245780	*SCG2, AP1S3, WDFY1, MRPL44, SERPINE2, FAM124B, CUL3*	
Rear Teat End Shape	26	50630351	***KNDC1****, SYCE1, TCERG1L, CYP2E1, LOC528422, LOC112444506, ADGRA1, LOC100850437, LOC101906198, LOC112444503, CFAP46, LOC112444502, LOC112444507, NKX6-2, INPP5A, LOC112444505*	morphology; immunology
Rear Teat Width[3]	25*	38568564*	*LOC618554, LOC101906717*	N/A
Rear Teat Width[4]	10		***CTDSPL2****, EIF3J, SPG11, LOC112448593, PATL2*	N/A
	11*	104129366*	*LOC112448855, LHX3, QSOX2, LOC787891, GPSM1, LOC101902280, DNLZ, CARD9, SNAPC4, ENTR1,*	morphology; immunology

		PMPCA, INPP5E, SEC16A, NOTCH1, LOC112448856, EGFL7, MIR126, LOC101902839, LOC101902895, FAM69B, LOC112448928, LOC107132967, LOC100848307, LOC112448857, ABO, LOC112448956, SURF6, LOC11244890, MED22, RPL7A, LOC100139115, LOC100112448907, LOC100112448908, LOC100112448904, LOC100112448903, LOC100112448905, SURF2, SURF4, STKLD1, LOC107132968, REXO4, ADAMTS13, CACFD1, SLC2A6, LOC112448858, TRNAC-GCA, MYMK, ADAMTSL2, FAM163B, DBH, SARDH, VAV2	
16	61802991-62196774	*CEP350, QSOX1, LOC112441858, LHX4, ACBD6, MIR669, XPR1, TRNAC-ACA, LOC107133256, KIAA1614, STX6, MR1, IER5, CACNA1E, LOC104974498, ZNF648,*	morphology; immunology
16*	63823597*	*LOC101905162, GLUL, TEDDM1, RGSL1, RNASEL, RGS16, RGS8, LOC101905664, NPL, DHX9, SHCBP1L, LAMC1, LAMC2, NMNAT2*	morphology; immunology
18	17655467	*LOC112442233, CBLN1, C18H16orf78, ZNF423, TRNAG-CCC*	N/A
18	20537778	*LOC516179, TOX3*	N/A
18	42468232	*MIR2899, LOC112442482, TRNAG-CCC, LOC617301, ZNF507, DPY19L3*	N/A
19	22640468	*VPS53, MIR2336, RFLNB, C19H17orf97, TRNAG-UCC, RPH3AL, LOC104975006, DOC2B, LOC112442619, YWHAE, TRNAE-UUC, CRK, MYO1C,* ***INPP5K****, PITPNA, SLC43A2, SCARF1, RILP, PRPF8, TLCD2, MIR22, WDR81, SMYD4, SERPINF1, RPA1, RTN4RL1, LOC112442621, DPH1, OVCA1, MIR132, MIR212, HIC1, SMG6, LOC112442776*	morphology; immunology
19	23978522-23997890	*RAP1GAP2, OR1D5, LOC101906737, LOC618593, OR1G1, LOC540082, LOC532238, LOC522582, LOC112442765,* ***LOC520835****, LOC59525, LOC59526*	N/A

	19	29058547-29063744	*GLP2R, RCVRN,* ***GAS7***	immunology
	25*	35208040*	*CUX1, TRNAW-CCA, MIR2388, LOC112444316, LOC112444338, MYL10, COL26A1, LOC104970468, IFT22, FIS1, PLOD3, LOC618076, NAT16, VGF, AP1S1, LOC101902751, SERPINE1, TRIM56, LOC101902689, LOC101909082, LOC107131854*	morphology; immunology
	25	40126743-40190566	***SDK1***	immunology
Udder Depth	5	113268242	***TCF20****, LOC104976976*	morphology
Udder Height	6	102964124-102982437	***LOC100298890***	N/A
	14	27024015	*CLVS1,* ***ASPH***	morphology
	15	15545765-15782913	***AMOTL1, LOC112441606***	N/A
	18	62273143-62481417	*TNNT1, PPP1R12C, LOC112442386, LOC112442387, EPS8L1,* ***RDH13****, LOC100848752, GP6, NLRP2, LOC100336589, LOC100852077,* ***LOC112442414***	morphology; immunology
	22	46733454	***CACNA2D3****, LOC112443534*	N/A
Udder Width	15	75722222	*PRDM11, LOC101906676, SYT13, LOC107133190, LOC112441655, CHST1, LOC104974324, LOC107133191, SLC35C1, CRY2, MAPK8IP1, C15H11orf94, PEX16, LARGE2, PHF21A, CREB3L1*	morphology; immunology
Front Teat Placement[5]	9	58002055-58079933	*LOC101902249, LOC112448054*	N/A
Udder Depth[5,6]	17	34476230-34552407	*SPRY1,* ***SPATA5****, LOC112442097, NUDT6, FGF2*	morphology

[1]Positions based on ARS_UCD 1.2

[2]Genes in LD with significantly associated SNP, or +/- 500 kb of associated SNP in case of no LD, bolded text indicates associated SNP is within the gene

[3]case-control GWA of narrow versus wide rear teats divided at the median value

[4]linear GWA of quantitative rear teat width scores
[5]primiparous only subset of cows (n = 144)
[6]not passing Bonferroni correction, QTL significantly associated at FDR < 0.05
