# Supplementary figures and images for: Direct Phenotyping and Principal Component Analysis of Type Traits Implicate Novel QTL in Bovine Mastitis through Genome-Wide Association
Source: Animals (Basel). 2021 Apr 17;11(4):1147. doi: 10.3390/ani11041147 (PMC8072530; doi:10.3390/ani11041147)

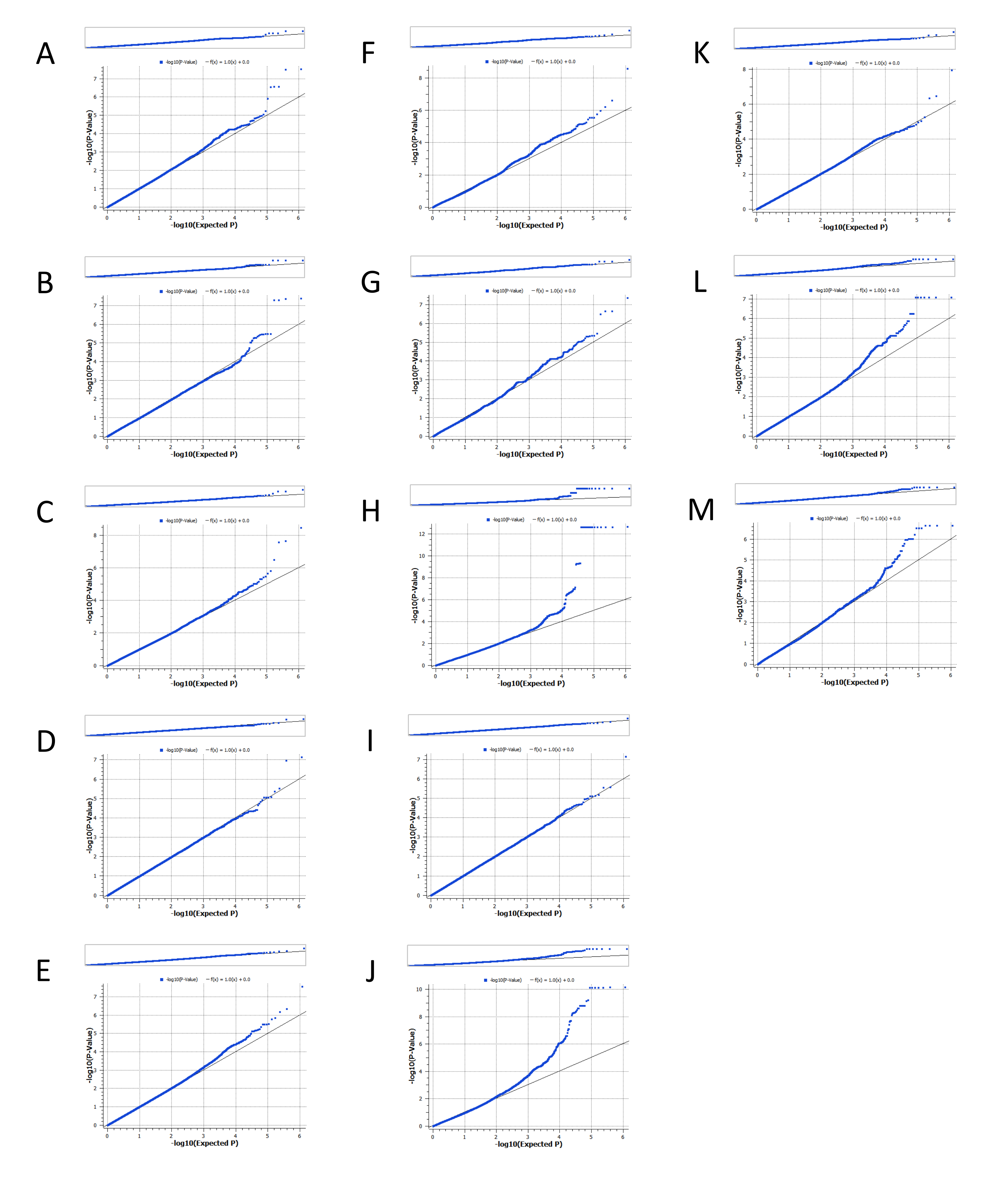

Supplement: Supplementary file 1 [file animals-11-01147-s001.zip › MilesA_Animals_SupplFig1.tif]

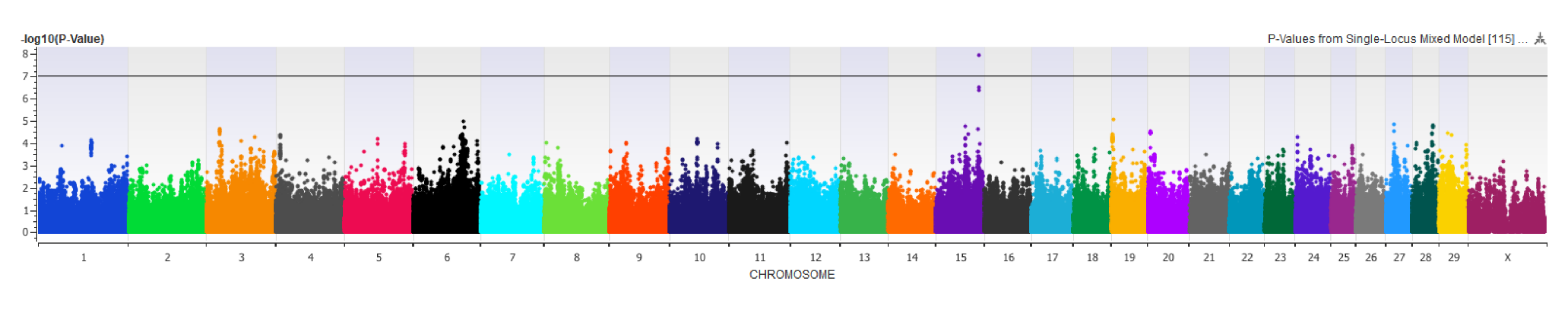

Supplement: Supplementary file 1 [file animals-11-01147-s001.zip › MilesA_Animals_SupplFig2.tif]

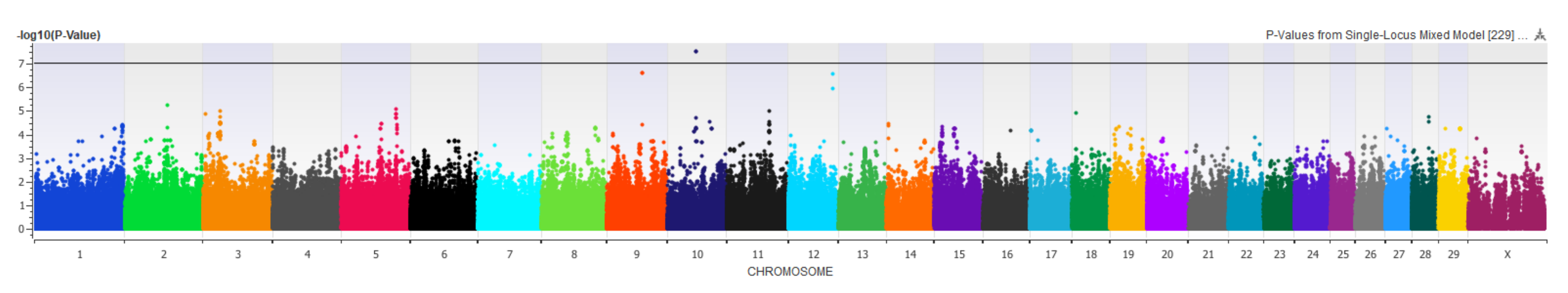

Supplement: Supplementary file 1 [file animals-11-01147-s001.zip › MilesA_Animals_SupplFig3.tif]

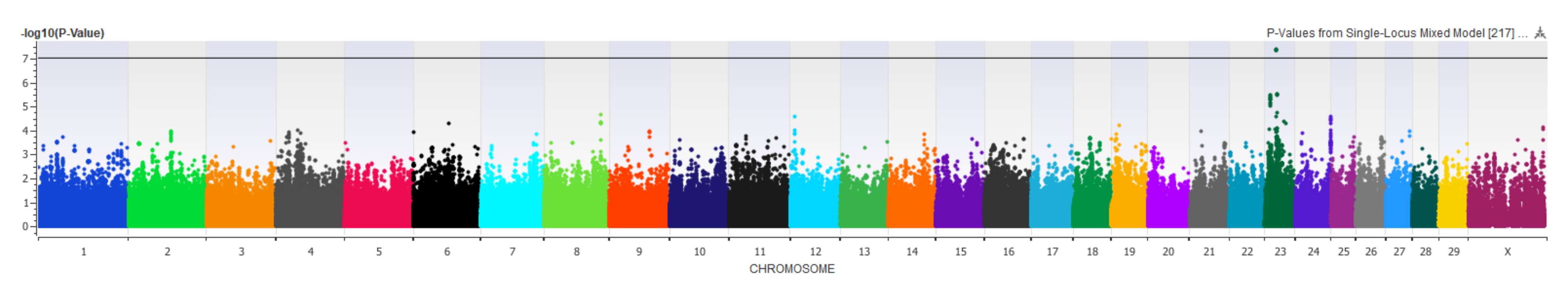

Supplement: Supplementary file 1 [file animals-11-01147-s001.zip › MilesA_Animals_SupplFig4.tif]

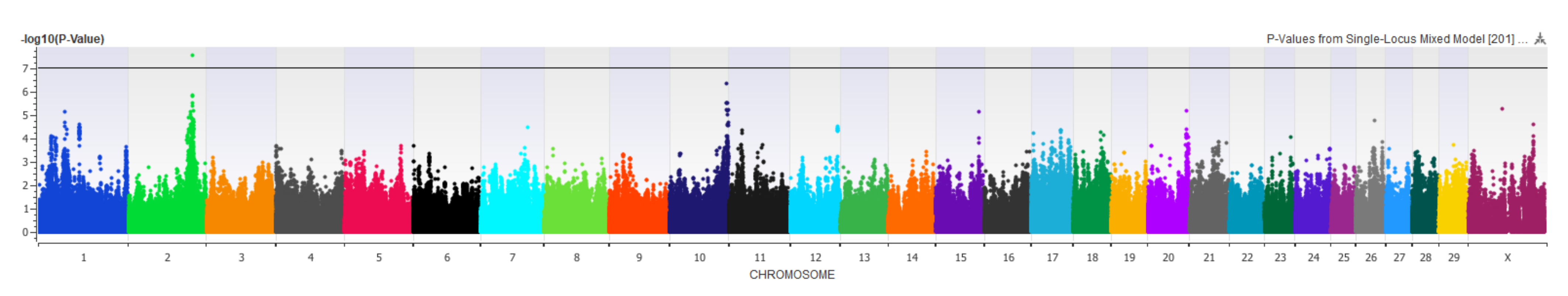

Supplement: Supplementary file 1 [file animals-11-01147-s001.zip › MilesA_Animals_SupplFig5.tif]

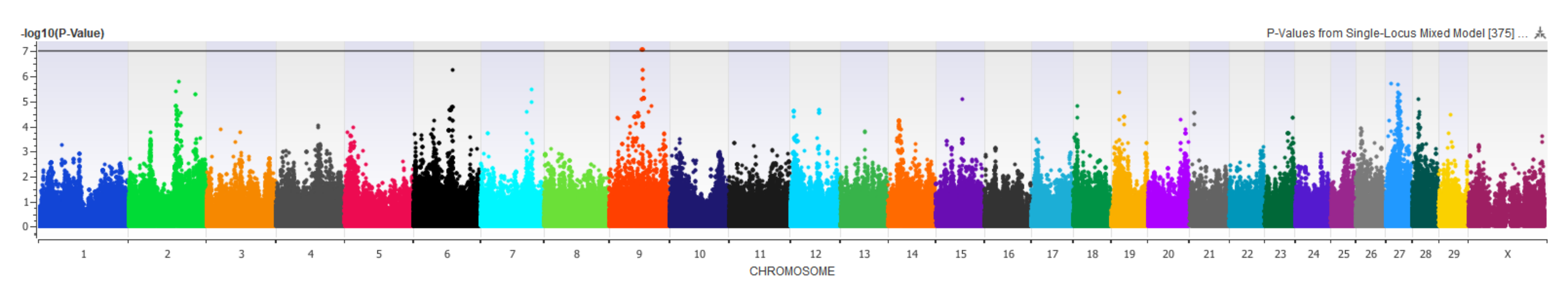

Supplement: Supplementary file 1 [file animals-11-01147-s001.zip › MilesA_Animals_SupplFig6.tif]
